# 2,4,5-trimethoxy-2′-trifluoromethylchalcone Attenuates Oxidative Stress and Inflammasome-Associated Inflammation in Experimental Models of Crystal-Induced Inflammation

**DOI:** 10.3390/antiox15091197

**Published:** 2026-09-18

**Authors:** Laura Catalán, Isabel García-Arnandis, María Carmen Terencio, María José Alcaraz, María Luisa Ferrándiz, María Carmen Montesinos, María Carmen Carceller

**Affiliations:** 1 Interuniversity Research Institute for Molecular Recognition and Technological Development (IDM), University of Valencia, Polytechnic University of Valencia, Av. Vicent Andrés Estellés 22, 46100 Burjassot, Valencia, Spain; lcatala8@xtec.cat (L.C.); isabel.garcia-arnandis@uv.es (I.G.-A.); carmen.terencio@uv.es (M.C.T.); maria.j.alcaraz@uv.es (M.J.A.); m.carmen.carceller@uv.es (M.C.C.); 2 Department of Pharmacology, Faculty of Pharmacy and Food Sciences, University of Valencia, Av. Vicent Andrés Estellés 22, 46100 Burjassot, Valencia, Spain

**Keywords:** 2,4,5-trimethoxy-2′-trifluoromethylchalcone, gouty arthritis, crystal-induced inflammation, inflammasome, caspase-1, oxidative stress, NF-κB, Nrf2, multi-target compound, pyroptosis

## Abstract

Crystal-induced arthropathies, including gout and calcium pyrophosphate deposition disease, are characterized by activation of inflammatory and oxidative pathways involving nuclear factor-kappa B (NF-κB) signaling, inflammasome activation, and reactive oxygen species (ROS) generation. This study evaluated the anti-inflammatory and antioxidant effects of 2,4,5-trimethoxy-2′-trifluoromethylchalcone (CH) in experimental models of crystal-induced inflammation and gouty arthritis. CH was evaluated in lipopolysaccharide (LPS)/adenosine 5′-triphosphate (ATP)-stimulated murine peritoneal macrophages and in murine models of CPPD crystal-induced air pouch inflammation and monosodium urate (MSU)-induced gouty arthritis. Cytokine production was determined by ELISA, whereas NF-κB and caspase-1 activation were evaluated by Western blot. ROS generation and nuclear translocation of nuclear factor erythroid 2-related factor 2 (Nrf2) were also assessed. CH reduced interleukin (IL)-1β, IL-18, tumor necrosis factor (TNF)-α, and IL-6 production in activated macrophages and attenuated lactate dehydrogenase release associated with pyroptosis. Mechanistically, CH reduced p65 NF-κB phosphorylation, caspase-1 activation, and ROS generation while promoting Nrf2 nuclear translocation. In vivo, CH reduced leukocyte infiltration, myeloperoxidase activity, inflammatory cytokine levels, and paw edema in CPPD- and MSU-induced models. These findings suggest that CH acts as a multitarget compound capable of simultaneously modulating inflammatory and oxidative pathways involved in crystal-induced arthropathies, which may represent a potential advantage over approaches targeting a single inflammatory pathway.

## 1. Introduction

Crystal-induced arthropathies, including gout and calcium pyrophosphate deposition disease, are among the most common inflammatory joint disorders. These disorders are characterized by the deposition of monosodium urate (MSU) or calcium pyrophosphate dihydrate (CPPD) crystals in joints and periarticular tissues, leading to acute inflammatory episodes associated with pain, swelling and tissue damage [1,2,3]. In gout, persistent hyperuricemia promotes the formation and deposition of MSU crystals, which trigger recurrent inflammatory responses that may eventually progress to chronic joint damage and tophus formation [4].

The inflammatory response induced by pathogenic crystals is mainly mediated by cells of the innate immune system, particularly macrophages and neutrophils. Following crystal phagocytosis, these cells activate intracellular signaling pathways that promote the release of pro-inflammatory mediators and the recruitment of additional inflammatory cells into the affected tissue. Among these mechanisms, activation of nuclear factor kappa B (NF-κB) plays a pivotal role by inducing the transcription of several inflammatory genes, including tumor necrosis factor alpha (TNF-α), interleukin (IL)-6 and the inactive precursors of IL-1β and IL-18 [5,6].

A key event in crystal-induced inflammation is the activation of the nucleotide-binding oligomerization domain (NOD)-like receptor family pyrin domain containing 3 (NLRP3) inflammasome, a cytosolic multiprotein complex involved in innate immune responses. Assembly of the NLRP3 inflammasome complex promotes caspase-1 cleavage and the consequent maturation and release of IL-1β and IL-18, two cytokines that play a central role in the amplification of inflammatory responses during gout attacks [5,7,8]. In addition, caspase-1 activation has been associated with pyroptosis, an inflammatory form of programmed cell death characterized by membrane disruption and release of intracellular mediators that further enhance inflammation [9].

Reactive oxygen species (ROS) also contribute significantly to the pathophysiology of crystal-induced inflammation. Crystal phagocytosis promotes oxidative stress through increased production of both extracellular ROS and mitochondrial-derived ROS, which has been closely associated with NLRP3 inflammasome activation and NF-κB signaling [10,11]. Excessive ROS generation not only amplifies inflammatory mediator production but may also contribute to tissue injury and persistence of inflammation. Conversely, activation of endogenous antioxidant pathways may exert protective effects under inflammatory conditions.

Among these antioxidant mechanisms, nuclear factor erythroid 2-related factor 2 (Nrf2) is considered a major regulator of cellular defense responses against oxidative stress. Under oxidative stress conditions, Nrf2 translocates into the nucleus and promotes the transcription of antioxidant and cytoprotective genes [12]. Increasing evidence suggests that Nrf2 activation may negatively regulate inflammatory signaling pathways, including NF-κB activation and NLRP3 inflammasome signaling, supporting the relevance of antioxidant strategies in inflammatory diseases such as gout [13].

Current therapeutic approaches for gout and related crystal-induced arthropathies are mainly based on anti-inflammatory agents, including nonsteroidal anti-inflammatory drugs, colchicine and glucocorticoids, as well as urate-lowering therapies for long-term management [14,15]. Although these treatments are effective in many patients, their use may be limited by adverse effects, contraindications or incomplete clinical responses, particularly in patients with comorbidities [3]. Therefore, the identification of novel compounds capable of simultaneously modulating inflammatory and oxidative pathways remains of considerable interest [16].

Chalcones are naturally occurring or synthetic flavonoid-related compounds that have shown anti-inflammatory and antioxidant properties in several experimental models [17,18]. Previous studies from our group demonstrated the anti-inflammatory activity of different chalcone derivatives through inhibition of neutrophil migration and inflammatory mediator production, including nitric oxide, prostaglandin E2 and tumor necrosis factor (TNF)-α, as well as suppression of NF-κB activation and inducible nitric oxide synthase expression [19,20,21]. In addition, fluorinated trimethoxychalcones have been associated with potent inhibition of nitric oxide production and anti-inflammatory activity both in activated macrophages and experimental arthritis models [22,23]. More recently, antioxidant and anti-inflammatory effects linked to modulation of ROS generation, inflammasome-associated pathways, NF-κB signaling and redox-sensitive mechanisms have also been described for several chalcone derivatives [18,24,25,26]. These mechanisms are particularly relevant to crystal-induced inflammation, in which neutrophil recruitment, oxidative stress, NF-κB activation and inflammasome signaling are closely interconnected and contribute to disease progression.

**Figure 1:**
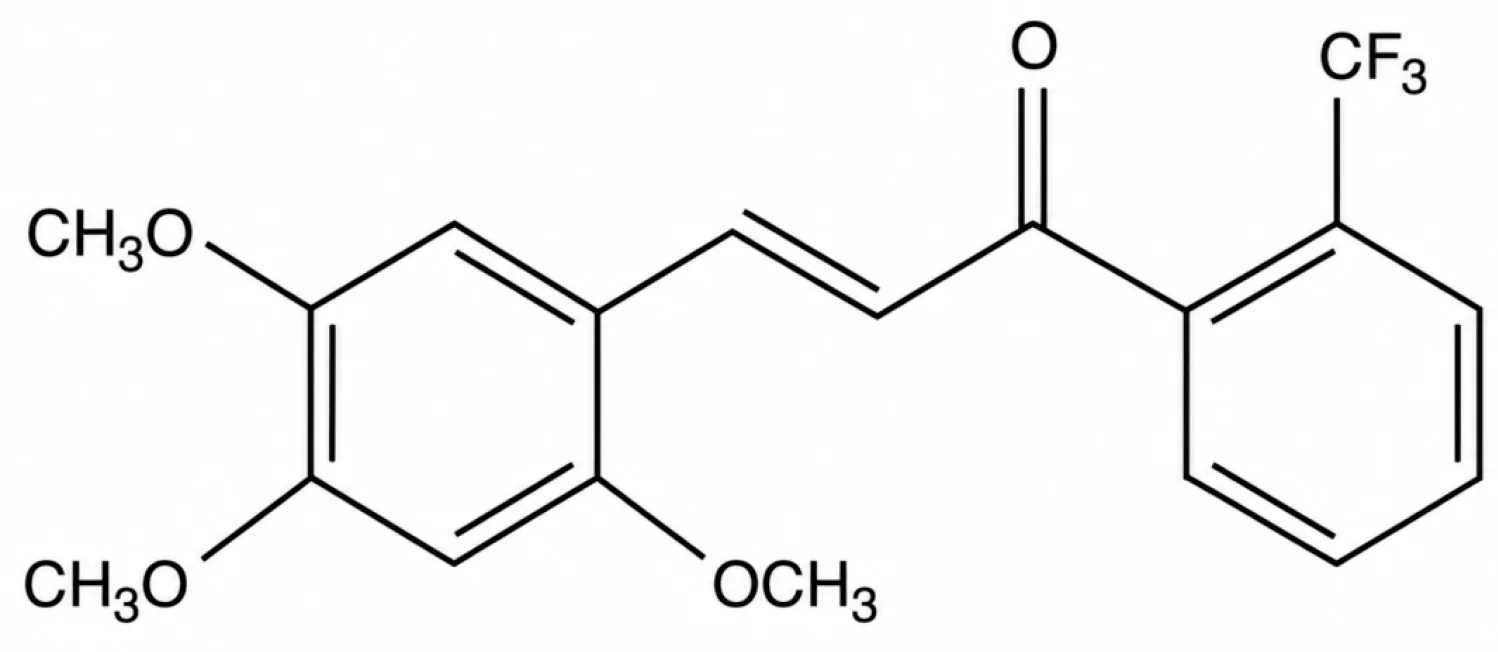
Chemical structure of the chalcone derivative 2,4,5-trimethoxy-2′-trifluoromethylchalcone (CH).

Given the close interplay between oxidative stress, inflammasome activation and NF-κB signaling in crystal-induced inflammation, compounds capable of simultaneously modulating these pathways may represent promising therapeutic strategies and may offer a potential advantage over approaches primarily targeting individual inflammatory mechanisms. Based on these observations, we hypothesized that 2,4,5-trimethoxy-2′-trifluoromethylchalcone (CH, Figure 1) could attenuate crystal-induced inflammatory responses through coordinated modulation of redox-sensitive and inflammasome-related pathways.

Accordingly, the present study was designed to evaluate the anti-inflammatory and antioxidant effects of CH in experimental models related to crystal-induced inflammation and gouty arthritis. For this purpose, its effects were analyzed in lipopolysaccharide (LPS)/adenosine 5′-triphosphate (ATP)-stimulated murine peritoneal macrophages, as well as in CPPD crystal-induced air pouch inflammation and MSU-induced gouty arthritis models in mice, with particular emphasis on ROS generation, NF-κB activation, caspase-1 signaling and Nrf2 nuclear translocation.

## 2. Materials and Methods

### 2.1. Study Compound

The 2,4,5-trimethoxy-2′-trifluoromethylchalcone (CH) under study (composition = C_19_H_17_O_4_F_3_; molecular weight = 366.3304 g/mol) was provided by Professor José Domínguez from the Organic Synthesis Laboratory of the Faculty of Pharmacy, Universidad Central de Venezuela [27].

### 2.2. Experimental Animals

The experiments were conducted using 10–12-week-old male C57BL/6 mice weighing 20–25 g (Charles River Laboratories, Écully, France). Animals were maintained under controlled environmental conditions, with a temperature of 22 ± 3 °C, relative humidity of 60% and a 12-h light/dark cycle. The animals were monitored and cared for by veterinarians and accredited animal facility staff throughout the experimental procedures. No mortality or overt treatment-related signs of toxicity were observed following CH administration at the doses used in the present study. All mice had free access to standard pellet diet and water ad libitum.

All in vivo studies were performed in accordance with EU Directive 2010/63/EU for animal experiments. Experimental protocols were approved by the Ethics Committee for Animal Experimentation of the University of Valencia and by the Valencian Government, Spain (2019/VSC/PEA/0137, 2017/VSC/PEA/00150 and 2017/VSC/PEA/00151).

### 2.3. Isolation and Culture of Peritoneal Macrophages

Peritoneal macrophages were elicited in C57BL/6 mice by intraperitoneal administration of 1 mL of 3% Brewer thioglycolate medium (Sigma-Aldrich, St. Louis, MO, USA) prepared in water. After 96 h, cells were collected by peritoneal lavage with 5 mL of phosphate-buffered saline (PBS; Gibco, Paisley, UK) and centrifuged at 400× *g* for 6 min. The resulting pellet was resuspended in Roswell Park Memorial Institute medium (RPMI 1640 medium; Biowest, Riverside, MO, USA) at 37 °C, and cells were seeded at a density of 2 × 10^6^ cells/mL in RPMI supplemented with 10% fetal bovine serum (FBS, Biowest, Riverside, MO, USA) and 1% penicillin/streptomycin (Gibco, Grand Island, NY, USA). Cultures were maintained at 37 °C in a humidified atmosphere containing 5% CO_2_ for 18 h.

Prior to treatment, the culture medium was refreshed. Cells were then exposed to CH, prepared as a 10^−2^ M stock solution in methanol and further diluted in culture medium to final concentrations of 1 µM and 10 µM. Corresponding vehicle controls received the same final concentration of methanol (<0.1%). After a 30 min preincubation period, lipopolysaccharide (LPS; Sigma-Aldrich, St. Louis, MO, USA) was added at a final concentration of 1 μg/mL for 4 h. Medium was then replaced with RPMI without FBS, and cells were stimulated with 5 mM adenosine 5′-triphosphate (ATP; Sigma-Aldrich, St. Louis, MO, USA) for 10 or 30 min. Experimental groups differed according to the assay. For MTT, LDH release, ROS generation, and Nrf2 nuclear translocation experiments, an additional group treated with CH alone at the highest concentration tested was included to evaluate potential intrinsic cytotoxic, antioxidant, or Nrf2-modulating effects independent of inflammatory stimulation. In contrast, cytokine production, NF-κB phosphorylation, and caspase-1 activation were assessed under inflammatory conditions, comparing stimulated control and CH-treated stimulated cells, since basal activation of these pathways is minimal in unstimulated macrophages.

### 2.4. Assessment of CH Cytotoxicity Using the MTT Assay

Peritoneal macrophages were seeded in 96-well plates (TC Plate 96 Well, Sarstedt, Nümbrecht, Germany) at a concentration of 10^6^ cells/mL (final volume 200 µL per well) and treated with LPS and ATP as described. After stimulation, cells were incubated with the yellow tetrazole salt, 3-(4,5-dimethylthiazol-2-yl)-2,5-diphenyl tetrazolium bromide (MTT; #Cat. M2128; Sigma-Aldrich, St. Louis, MO, USA), at a final concentration of 200 µg/mL in culture medium for 1 h under the usual conditions. The medium was then removed and 100 µL of dimethyl sulfoxide was added to solubilize the resulting formazan crystals [28]. Absorbance was determined using a VICTOR3™ V1420 spectrophotometer (PerkinElmer, Turku, Finland) at a wavelength of 531 nm. Cell viability was expressed as a percentage relative to non-stimulated control cells, which were defined as 100% viability (0% cytotoxicity).

### 2.5. Assessment of Pyroptosis by Lactate Dehydrogenase (LDH) Assay

Cell death associated with caspase-1-dependent pyroptosis was determined by quantifying lactate dehydrogenase (LDH) release in the supernatants of elicited macrophages treated with CH in the absence or presence of LPS (1 μg/mL) plus ATP (5 mM). For the assay, 50 µL of supernatant was transferred to a clear 96-well plate and mixed with an equal volume of reaction buffer containing 0.2 M Tris-HCl (pH 7.2), β-nicotinamide adenine dinucleotide (β-NAD, 250 µg; Sigma-Aldrich, St. Louis, MO, USA), lactic acid (1.2 mg; Sigma-Aldrich), MTT (130 µg; Sigma-Aldrich), and phenazine methosulfate (PMS; 30 µg; Sigma-Aldrich). Plates were incubated at room temperature in the dark, and absorbance was recorded at 550 nm after 90 min using a VICTOR3™ V1420 plate reader (PerkinElmer, Turku, Finland).

### 2.6. Enzyme-Linked Immunosorbent Assay (ELISA)

Elicited macrophages were seeded in 6-well plates at a density of 1 × 10^6^ cells/mL and treated with CH in the absence or presence of LPS (1 μg/mL) plus ATP (5 mM). The concentrations of TNF-α, IL-1β, IL-18, IL-6, and CXCL-1 in culture supernatants were determined using commercial sandwich ELISA kits, following the manufacturers’ protocols. The detection limits were 31.3 pg/mL for TNF-α (#Cat. DY410-05; R&D Systems, Minneapolis, MN, USA), 15.6 pg/mL for IL-1β (#Cat. DY401-05; R&D Systems), 19.0 pg/mL for IL-18 (#Cat. BMS618-3; Thermo Fisher Scientific, Göteborg, Sweden), 4.0 pg/mL for IL-6 (#Cat. 88-7064-88; Thermo Fisher Scientific, Waltham, MA, USA), and 4.0 pg/mL for CXCL-1 (#Cat. DY453-05; R&D Systems). Absorbance was measured using a Wallac 1420 VICTOR3™ microplate reader (PerkinElmer, Turku, Finland).

### 2.7. Determination of Caspase-1 and NF-κB Activation by Western Blot

Protein concentrations in supernatants and cell lysates from peritoneal macrophages, air-pouch exudates, and paw homogenates were quantified using the DC Bio-Rad Protein Assay Kit (Bio-Rad, Hercules, CA, USA). Supernatants were used to determine the active p20 fragment of caspase-1 in macrophages and air-pouch samples. Adherent macrophages were lysed in 500 μL of lysis buffer, and after centrifugation (10,000× *g*, 10 min, 4 °C), the resulting supernatants were used to evaluate the expression of procaspase-1 (p48) and phosphorylated p65 NF-κB.

Protein samples (10–20 μg per lane) were separated by sodium dodecyl sulfate–polyacrylamide gel electrophoresis (SDS-PAGE) (12.5% gels for caspase-1 and 10% for NF-κB) and transferred onto polyvinylidene difluoride (PVDF) membranes (GE Healthcare Life Sciences, Barcelona, Spain). Membranes were blocked with 3% (*w*/*v*) non-fat dry milk and incubated overnight at 4 °C with primary antibodies against caspase-1 (p48 and p20; 1:2000; #Cat. AG-20B-0042, AdipoGen Life Science, Liestal, Switzerland) and phosphorylated p65 NF-κB (p-p65 NFκB, Ser 536) (1:100; #Cat. 3031, Cell Signaling Technology, Beverly, MA, USA).

Subsequently, membranes were incubated for 1 h at room temperature with peroxidase-conjugated secondary antibodies: goat anti-rabbit IgG (1:5000; #Cat. P0448, Dako, Glostrup, Denmark) for p-p65 NF-κB, and goat anti-mouse IgG (1:4000; #Cat. A4416, Sigma-Aldrich, St. Louis, MO, USA) for caspase-1. β-actin (1:500; #Cat. A2066, Sigma-Aldrich) or glyceraldehyde-3-phosphate dehydrogenase (GAPDH) (1:2500; #Cat. G9545, Sigma-Aldrich) served as loading controls.

Immunoreactive bands were detected using enhanced chemiluminescence (ECL; RPN2232, Amersham, GE Healthcare Life Sciences, Barcelona, Spain) and visualized with an AutoChemi System (P/N97-0150-02) coupled to LabWorks v4.6 software (UVP Inc., Upland, CA, USA). Band intensities were quantified by optical densitometry using the Fiji downloads (Windows 32-bit) platform, an image-processing package distribution of ImageJ2 as previously described in detail [29].

### 2.8. Determination of Reactive Oxygen Species (ROS) Production

ROS production was determined at both mitochondrial and extracellular levels. Mitochondrial superoxide production was evaluated by fluorescence microscopy using the mitochondrial probe MitoSOX™ Red (Molecular Probes™, Invitrogen, Paisley, UK). Peritoneal macrophages were seeded onto 8-well Chamber Slide™ culture chambers (Nunc–Thermo Fisher, Rochester, NY, USA) at a density of 0.5 × 10^6^ cells/well in a final volume of 500 µL. After the adhesion period, cells were treated with CH and stimulated with LPS and ATP under the experimental conditions described above. Culture medium was removed and cells were incubated with 5 µM MitoSOX™ diluted in Hank’s Balanced Salt Solution (HBSS) containing Ca^2+^ and Mg^2+^ for 10 min at 37 °C. Cells were then washed three times with warm PBS and fixed with 4% paraformaldehyde for 15 min. After additional washes, slides were mounted using ProLong™ Gold Antifade Mountant with DAPI (Molecular Probes™, Invitrogen, Paisley, UK). Fluorescence images were acquired using an Olympus FV1000 confocal microscope (Olympus, Waltham, MA, USA).

Extracellular ROS generation was assessed by luminol-enhanced chemiluminescence. Peritoneal macrophages were seeded into black 96-well plates (Nunc–Thermo Fisher, Rochester, NY, USA) at a density of 1 × 10^6^ cells/mL in a final volume of 200 µL. Following the adhesion period, culture medium was replaced with HBSS containing Ca^2+^ and Mg^2+^. Cells were pretreated with CH for 30 min and stimulated with 12-O-tetradecanoylphorbol-13-acetate (TPA, 10^−6^ M) for 20 min in the presence of luminol (40 µM). Chemiluminescence was measured using a Wallac 1420 VICTOR3™ plate reader (PerkinElmer, Turku, Finland).

### 2.9. Determination of Nrf2 Translocation to the Nucleus by Immunofluorescence

Peritoneal macrophages were seeded in 8-well Chamber Slide™ slides at a concentration of 0.5 × 10^6^ cells/well in a final volume of 500 µL. After treatment with CH and stimulation with LPS and ATP, cells were washed twice with ice-cold PBS. The cells were then fixed with methanol at 4 °C for 15 min, washed again with PBS, and incubated overnight at 4 °C with anti-Nrf2 antibody (#Cat. ab137550; Abcam, Cambridge, UK) at a 1:800 dilution in PBS/BSA 1%. Following washing, the cells were incubated with Alexa Fluor^®^ 488 goat anti-rabbit secondary antibody (#Cat. A11008; Molecular ProbesTM Invitrogen, Paisley, UK) at a 1:500 dilution in PBS/BSA 1% for 1 h at room temperature. After additional washes, the chambers were removed and a drop of ProLong™ Gold Antifade Mountant with DAPI (#Cat. P36935; Molecular Probes™, Invitrogen, Paisley, UK) was added. Slides were mounted with coverslips and allowed to cure for 18 h before imaging. Fluorescence images were acquired using an Olympus FV1000 confocal microscope (Olympus, Waltham, MA, USA).

### 2.10. Mouse Air Pouch Model Induced by Calcium Pyrophosphate Dihydrate (CPPD) Crystals

A subcutaneous air pouch was generated by injecting 10 mL of sterile air into the dorsal region of the animals. After 3 days, the pouch was reinflated with an additional 5 mL of sterile air. On day 6 after the initial injection, CH, dissolved in physiological saline, was administered directly into the pouch at a concentration of 100 µg/pouch. Twenty minutes later, 1 mL of CPPD crystals (#Cat. tlrl-cppd; InvivoGen, San Diego, CA, USA), prepared at 1 mg/mL in sterile PBS, was injected into both control and treated groups, whereas the blank group received 1 mL of sterile PBS alone. Six hours after CPPD injection, mice were anesthetized and euthanized by cervical dislocation. Air-pouch exudates were collected and cell infiltration was quantified using a cell counter (Beckman Coulter™ Z2 Coulter^®^, Indianapolis, IN, USA). Exudates were centrifuged at 1200× *g* for 10 min at 4 °C and the supernatants were collected for the quantification of IL-1β, IL-18, TNF-α, CXCL-1, and IL-6 levels by ELISA, as well as for the analysis of p20 caspase-1 expression by Western blot and myeloperoxidase activity. The corresponding cell pellets were lysed and used to assess p48 caspase-1 and phosphorylated p65 NF-κB levels by Western blotting.

### 2.11. Determination of Myeloperoxidase Activity

Air-pouch exudate supernatants were incubated with PBS (pH 7.4) and phosphate buffer pH 5.4 (Na_2_HPO_4_ 0.09%, NaH_2_PO_4_ 1.15%) in the presence of hydrogen peroxide (0.05%) for 5 min. The reaction substrate 3, 3′, 5, 5′-tetramethylbenzidine (TMB) at 18 mM dissolved in dimethylformamide (prepared at 8% in distilled water) was then added and the plate was incubated for 3 min at 37 °C, stopping the reaction with 2 N sulfuric acid. Absorbance was quantified using a VICTOR3™ Wallac 1420 spectrophotometer (PerkinElmer, Turku, Finland) at a wavelength of 450 nm.

### 2.12. Mouse Gouty Arthritis Induced by Monosodium Urate (MSU) Crystals

CH was dissolved in physiological saline and administered orally at a dose of 30 mg/kg. This dose was selected based on previous studies demonstrating anti-inflammatory efficacy of chalcone derivatives at 30 mg/kg in experimental MSU-induced gouty arthritis models [30,31]. One hour later, 2 mg of MSU crystals (#Cat. tlrl-msu-25; InvivoGen, Toulouse, France), resuspended in 50 µL PBS, were injected subcutaneously into the plantar aponeurosis of the right paw. Paw edema was measured at 1,3, 6 and 24 h post injection using a digital plethysmometer (Digital Water Plethysmometer, Panlab S.L.U., Barcelona, Spain). At 24 h, animals were anesthetized and euthanized by cervical dislocation and the paws were excised and stored at −80 °C for subsequent homogenization in liquid nitrogen and downstream analyses.

### 2.13. Sample Collection and Processing

Frozen paw samples (−80 °C) were transferred to liquid nitrogen and homogenized in 1 mL of lysis buffer A (10 mM HEPES, pH 8.0; 1 mM EDTA; 1 mM EGTA; 10 mM KCl, 1 mM DTT, 5 mM NaF, 1 mM Na_3_VO_4_, 10 mM Na_2_MoO_4_, 1 µg/mL leupeptin, 0.1 µg/mL aprotinin, and 0.5 mM phenylmethylsulfonyl fluoride).

Samples were sonicated on ice (three cycles of 10 s), incubated for 10 min at 4 °C, and centrifuged at 1500× *g* for 5 min at 4 °C. The supernatants were collected and subjected to a second centrifugation (10,000× *g*, 5 min, 4 °C). Final supernatants were stored for subsequent determination of cytokine levels (IL-1β, TNF-α, IL-18, IL-6, and CXCL-1) by ELISA, measurement of myeloperoxidase activity, and analysis of protein expression (caspase-1 p20 and p48, and phosphorylated p65 NF-κB) by Western blotting.

### 2.14. Statistical Analysis

Data were analyzed using GraphPad Prism 7.0 (GraphPad Software Inc., La Jolla, CA, USA). Results are presented as mean ± standard deviation (SD).

Statistical significance was assessed using one-way analysis of variance (ANOVA) followed by Tukey’s post hoc test for multiple comparisons, including all pairwise group comparisons. For paw edema measurements, data were analyzed by two-way ANOVA (treatment × time) followed by Bonferroni’s post hoc test for multiple comparisons. Differences were considered statistically significant at *p* < 0.05.

## 3. Results

### 3.1. Effects of CH on Peritoneal Macrophages

#### 3.1.1. Effect of CH on Peritoneal Macrophages Viability

**Figure 2:**
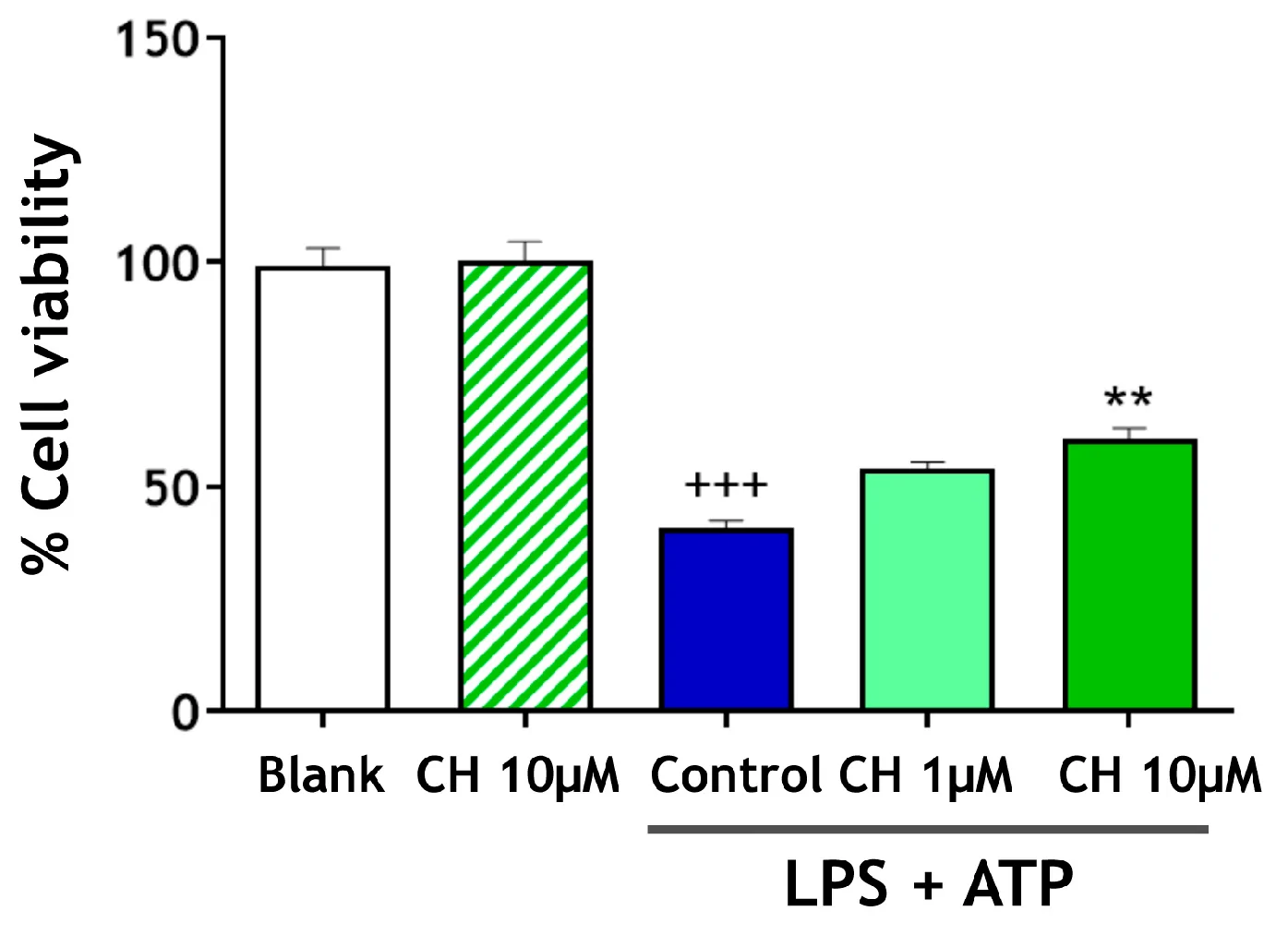
Effect of CH on cell viability in peritoneal macrophages stimulated with LPS and ATP. Macrophages were treated with CH in the absence or presence of LPS/ATP stimulation and cell viability was assessed by the MTT assay. Results are expressed by assigning 100% viability to the mean absorbance of the blank group. Data are presented as mean ± SD (*n* = 6). +++ *p* < 0.001 versus the blank group; ** *p* < 0.01 versus the control group (one-way ANOVA followed by Tukey’s test). CH: 2,4,5-trimethoxy-2′-trifluoromethylchalcone; LPS: lipopolysaccharide; ATP: adenosine triphosphate.

Cell viability was evaluated by the MTT assay in peritoneal macrophages stimulated with LPS and ATP. Under basal conditions, CH did not modify cell viability compared with the blank group, indicating the absence of cytotoxic effects at the concentrations tested. In contrast, stimulation with LPS/ATP significantly reduced cell viability. Treatment with CH partially reversed the reduction in MTT reduction capacity induced by LPS/ATP stimulation at both concentrations tested (Figure 2).

#### 3.1.2. Effect of CH on Pro-Inflammatory Cytokines Production

**Figure 3:**
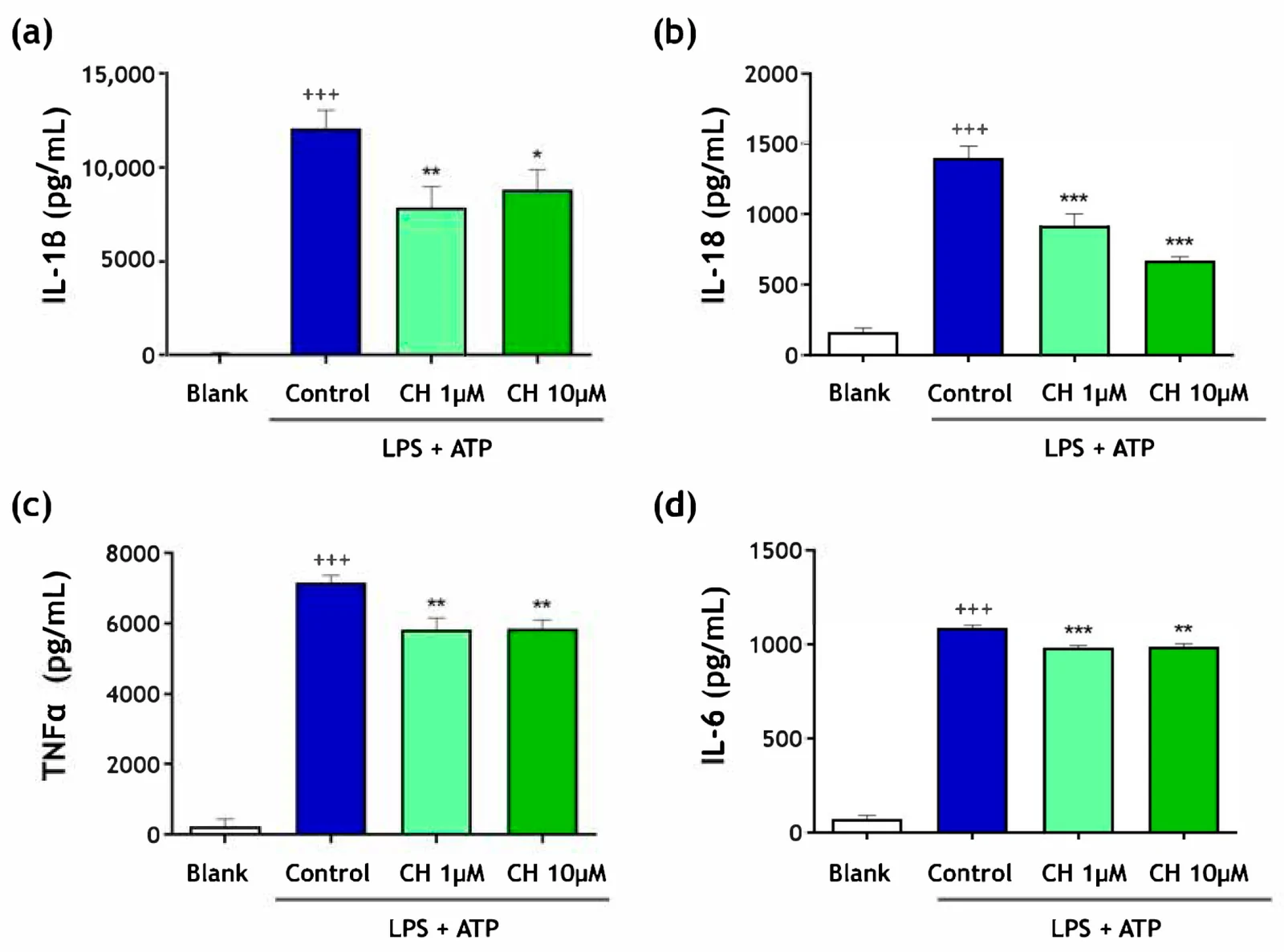
Effect of CH on pro-inflammatory cytokine release in peritoneal macrophages. Macrophages were treated with CH in the absence or presence of LPS/ATP stimulation, and cytokine levels were determined in culture supernatants by ELISA. (**a**) IL-1β; (**b**) IL-18; (**c**) TNF-α; (**d**) IL-6. Results are expressed as mean ± SD (*n* = 6). +++ *p* < 0.001 versus the blank group; * *p* < 0.05, ** *p* < 0.01, *** *p* < 0.001 versus the control group (one-way ANOVA followed by Tukey’s test). CH: 2,4,5-trimethoxy-2′-trifluoromethylchalcone; LPS: lipopolysaccharide; ATP: adenosine triphosphate.

The effect of CH on the production of pro-inflammatory cytokines was assessed by ELISA in the supernatants of peritoneal macrophages in presence or absence of LPS/ATP stimulus. As expected, stimulation with LPS/ATP markedly increased the production of all cytokines analyzed compared with basal conditions, whereas CH significantly reduced the release of IL-1β, IL-18, TNF-α, and IL-6 at both concentrations tested (Figure 3). The inhibitory effect was particularly evident at 10 µM, where IL-1β and IL-18 levels were reduced by 35% and 59%, respectively (Figure 3a,b).

#### 3.1.3. Effect of CH on NF-κB Activation

**Figure 4:**
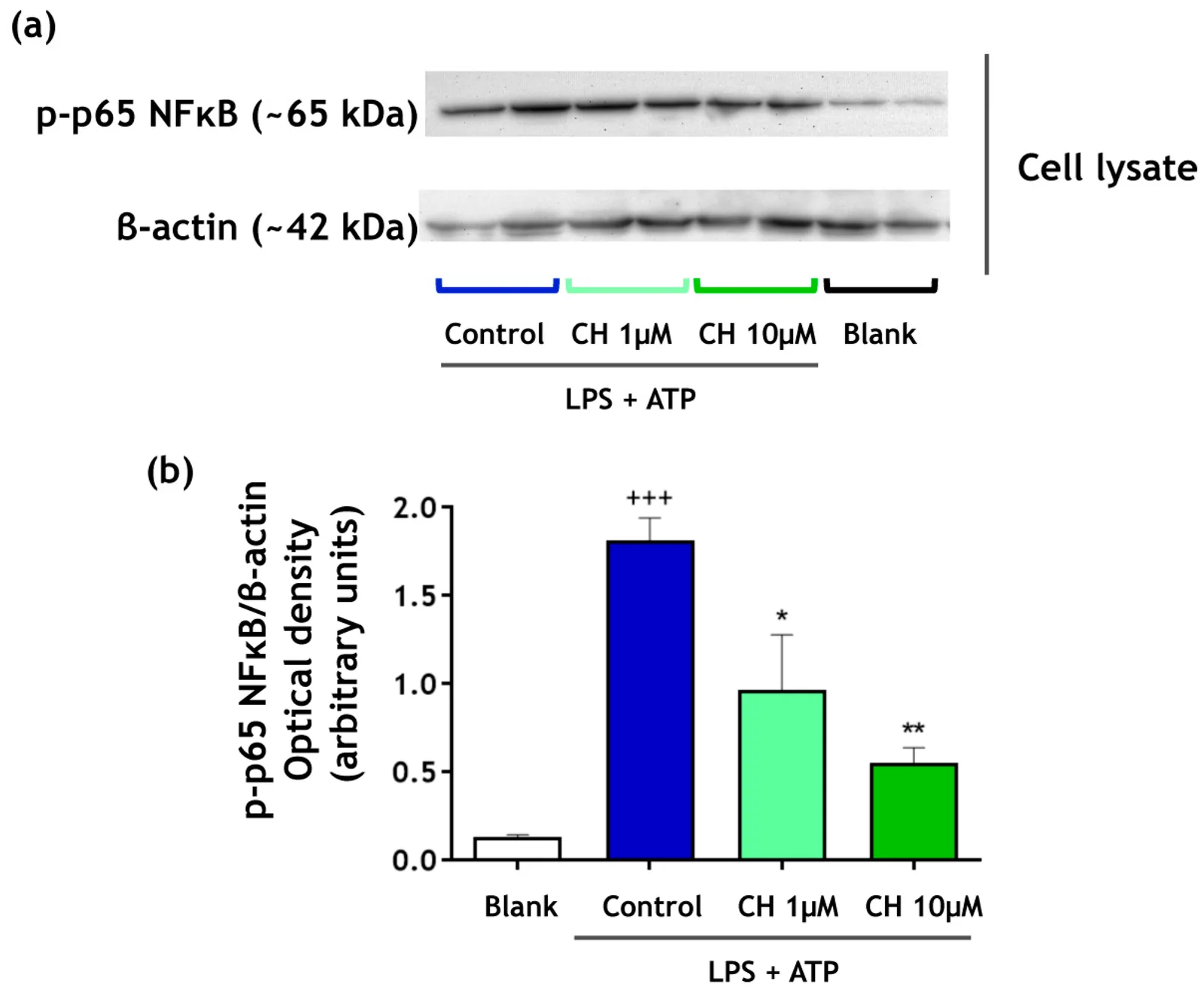
Effect of CH on phosphorylation of the p65 subunit of NF-κB in peritoneal macrophages stimulated with LPS and ATP. (**a**) Representative Western blot showing phosphorylated p65 NF-κB in macrophage lysates. β-actin is the loading control. (**b**) Densitometric analysis of optical density from all images obtained, expressed in arbitrary units as the ratio of phosphorylated p65 NF-κB subunit to β-actin. Results are expressed as mean ± SD (*n* = 4–5). +++ *p* < 0.001 versus the blank group; * *p* < 0.05, ** *p* < 0.01 versus the control group (one-way ANOVA followed by Tukey’s test). CH: 2,4,5-trimethoxy-2′-trifluoromethylchalcone; LPS: lipopolysaccharide; ATP: adenosine triphosphate.

Since NF-κB activation plays a central role in the inflammatory response, we investigated whether the inhibitory effects of CH on cytokine production could be associated with modulation of this pathway. To evaluate NF-κB activation, the phosphorylated form of the p65 NF-κB subunit (p-p65) was analyzed in cell lysates by Western blot. Phosphorylation of p65 facilitates its nuclear translocation and subsequent induction of pro-inflammatory gene expression [6]. Our data revealed low basal levels of p-p65 in unstimulated macrophages, while LPS/ATP treatment induced a marked increase in p65 phosphorylation. CH treatment markedly reduced p65 phosphorylation compared with stimulated control cells, indicating that CH attenuated NF-κB activation induced by LPS/ATP stimulation (Figure 4).

#### 3.1.4. Effects of CH on Caspase-1 Activation and LDH Levels

Since caspase-1 mediates the maturation of IL-1β and IL-18, we investigated whether the inhibitory effect of CH on these cytokines was associated with modulation of caspase-1 activation, which is characterized by cleavage of procaspase-1 and release of the active p20 subunit into the extracellular space. For this purpose, the active p20 subunit released into the supernatant was analyzed by Western blot and compared with intracellular procaspase-1 (p48) levels.

**Figure 5:**
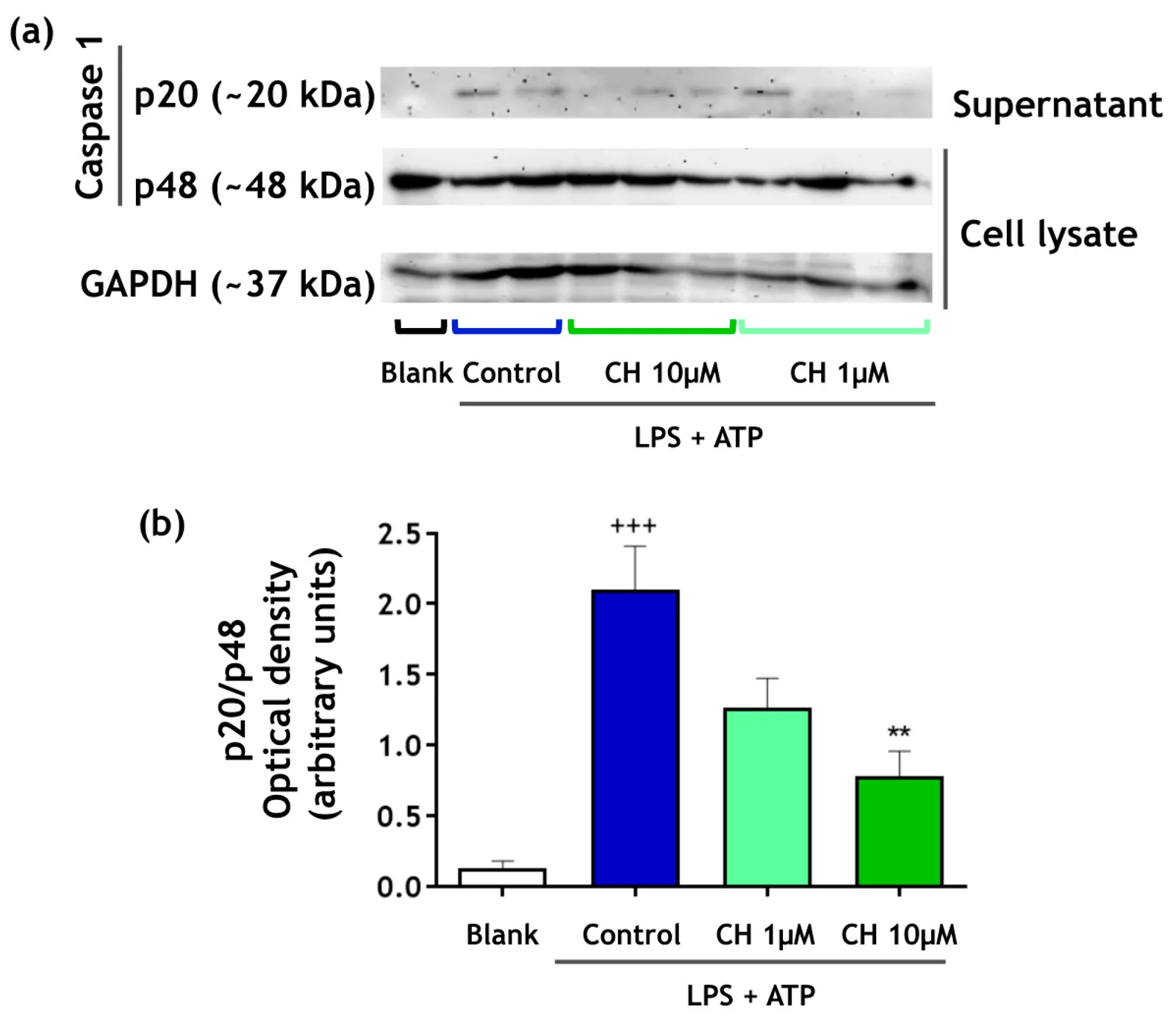
Effect of CH on caspase-1 activation in peritoneal macrophages stimulated with LPS and ATP. (**a**) Representative Western blot image showing the active caspase-1 (p20 subunit) and the pro-caspase-1 (p48 subunit) detected in the supernatant and cell lysate, respectively. GAPDH was used as the loading control. (**b**) Densitometric analysis of optical density expressed in arbitrary units as the ratio between the active p20 subunit and procaspase-1 p48. Results are presented as mean ± SD (*n* = 4–5). +++ *p* < 0.001 versus the blank group; ** *p* < 0.01 versus the control group (one-way ANOVA followed by Tukey’s test). CH: 2,4,5-trimethoxy-2′-trifluoromethylchalcone; LPS: lipopolysaccharide; ATP: adenosine triphosphate.

Figure 5a shows that stimulation with LPS and ATP markedly increased the release of the active p20 subunit in control cells compared with blank cells. Treatment with CH reduced the intensity of the p20 signal, consistent with inhibition of caspase-1 activation. Accordingly, analysis of the p20/p48 ratio confirmed a significant reduction in CH-treated cells compared with the control group (Figure 5b).

**Figure 6:**
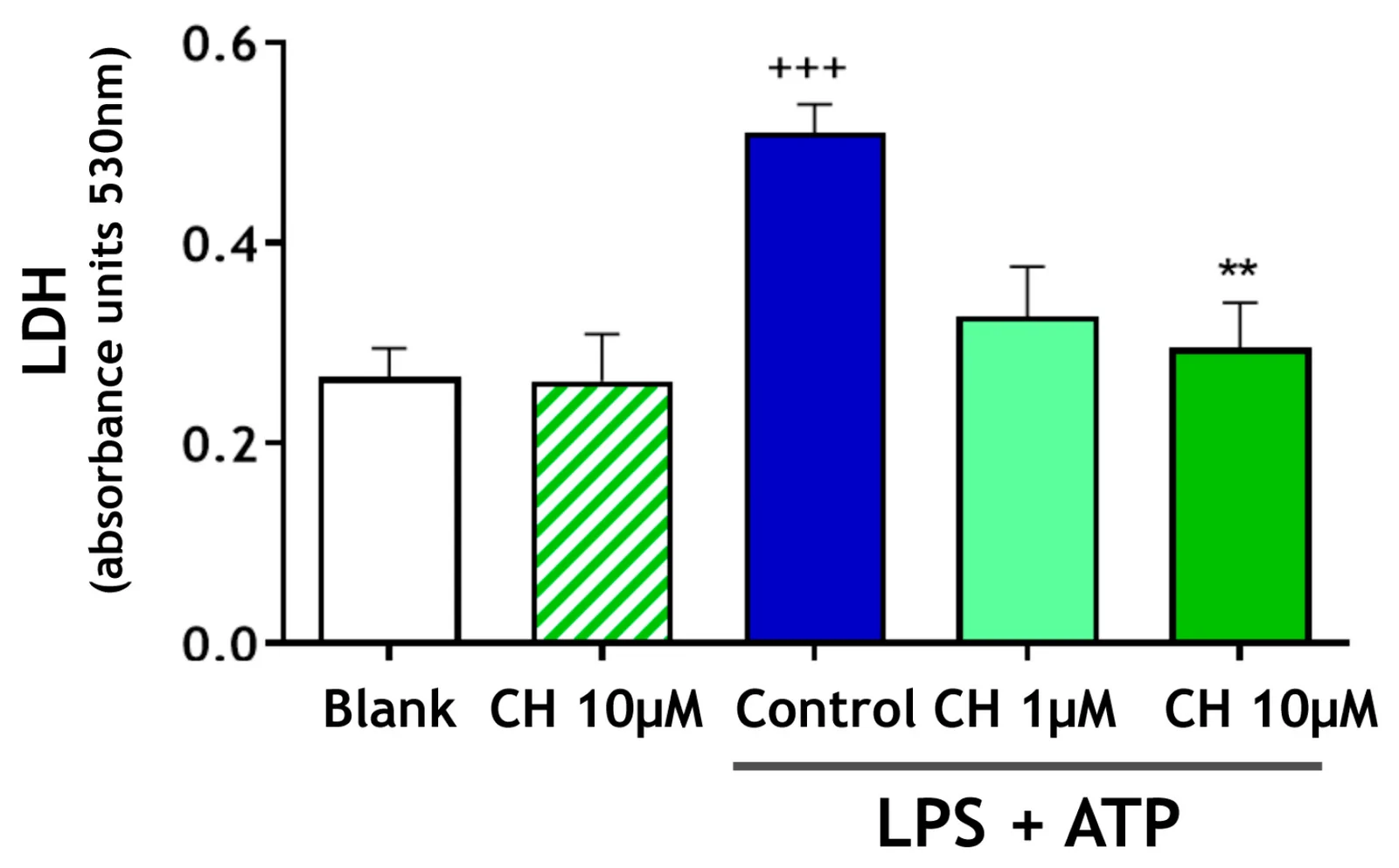
Effect of CH on LDH release in peritoneal macrophages stimulated with LPS and ATP. LDH activity levels are expressed as absorbance units at 530 nm. Results are expressed as mean ± SD (*n* = 6). +++ *p* < 0.001 versus the blank group; ** *p* < 0.01 versus the control group (one-way ANOVA followed by Tukey’s test). CH: 2,4,5-trimethoxy-2′-trifluoromethylchalcone; LDH: lactate dehydrogenase; LPS: lipopolysaccharide; ATP: adenosine triphosphate.

LDH activity was then evaluated in culture supernatants as an indicator of membrane damage associated with pyroptotic cell death. Consistent with its inhibitory effect on caspase-1 activation, CH significantly attenuated LDH release induced by LPS/ATP stimulation, suggesting reduced membrane damage associated with pyroptotic cell death. Treatment with CH alone did not modify LDH levels compared with unstimulated cells (Figure 6).

#### 3.1.5. Effect of CH on ROS Generation

ROS are involved in the regulation of inflammatory responses and have been closely associated with NLRP3 inflammasome activation in gout-related inflammation [10,11]. To investigate whether the anti-inflammatory effects of CH were associated with modulation of oxidative stress, extracellular and mitochondrial ROS production were evaluated in stimulated macrophages.

**Figure 7:**
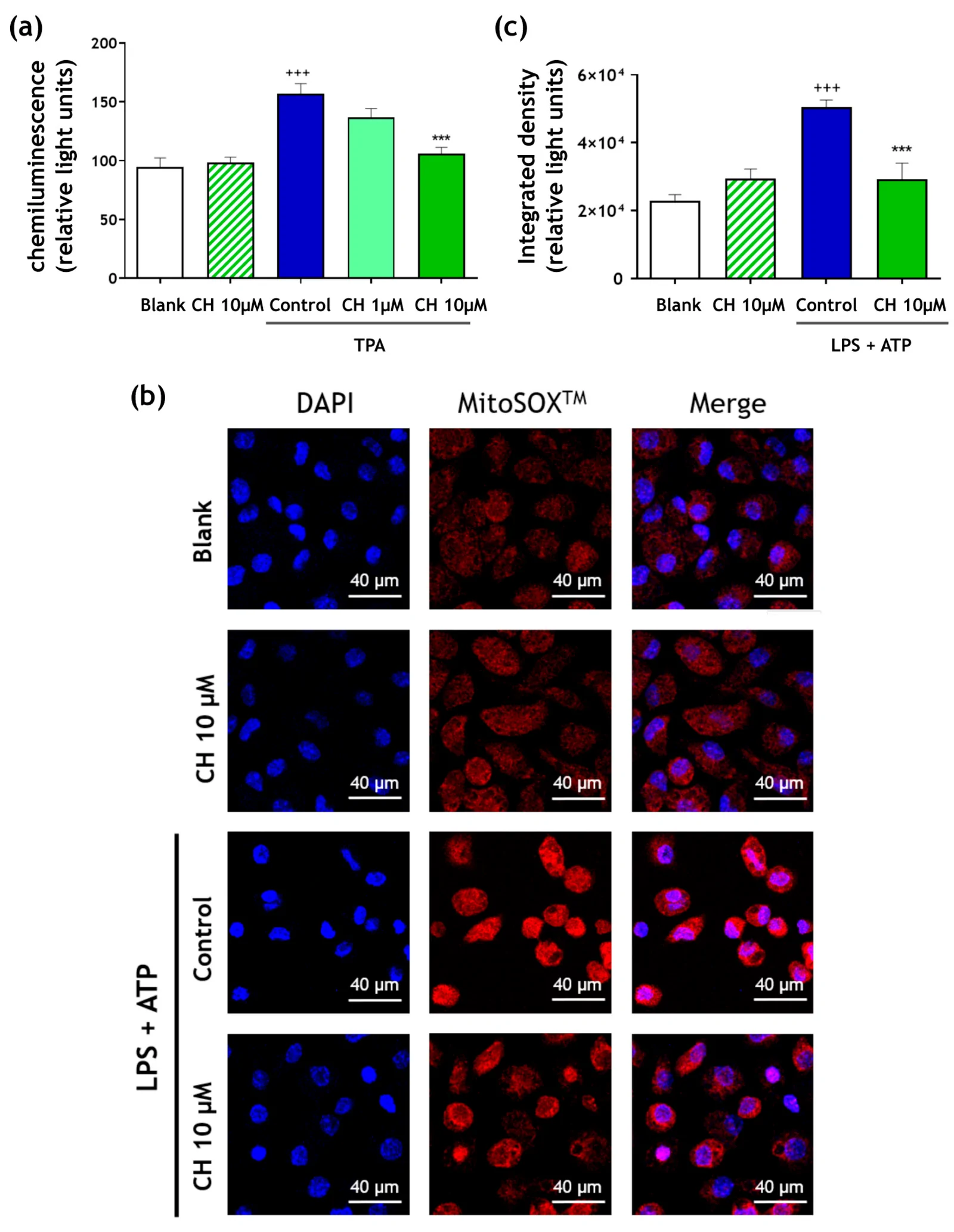
Effect of CH on extracellular and mitochondrial ROS production in peritoneal macrophages. (**a**) Extracellular ROS generation induced by TPA was determined by luminol-enhanced chemiluminescence and expressed as relative light units (RLU). (**b**) Representative MitoSOX™ fluorescence images showing mitochondrial ROS production in macrophages stimulated with LPS/ATP acquired using an Olympus FV1000 confocal microscope. Red fluorescence corresponds to MitoSOX™ staining and blue fluorescence corresponds to DAPI-stained nuclei. Scale bar: 40 μm. (**c**) Quantification of MitoSOX™ fluorescence intensity. Results are expressed as mean ± SD (*n* = 6). +++ *p* < 0.001 versus the blank group; *** *p* < 0.001 versus the control group (one-way ANOVA followed by Tukey’s test). CH: 2,4,5-trimethoxy-2′-trifluoromethylchalcone; LPS: lipopolysaccharide; TPA: 12-O-tetradecanoylphorbol-13-acetate; LPS: lipopolysaccharide; ATP: adenosine triphosphate.

Extracellular ROS generation, measured by luminol chemiluminescence, increased markedly after TPA stimulation. In contrast, lower ROS levels were detected in macrophages treated with CH (Figure 7a). On the other hand, mitochondrial ROS production was analyzed using the fluorescent probe MitoSOX™. Confocal microscopy revealed increased fluorescence intensity in LPS + ATP stimulated cells, indicating enhanced mitochondrial superoxide generation. This signal was visibly lower in CH-treated macrophages, which was confirmed by fluorescence quantification (Figure 7b,c).

#### 3.1.6. Effect of CH on Nrf2 Nuclear Translocation

**Figure 8:**
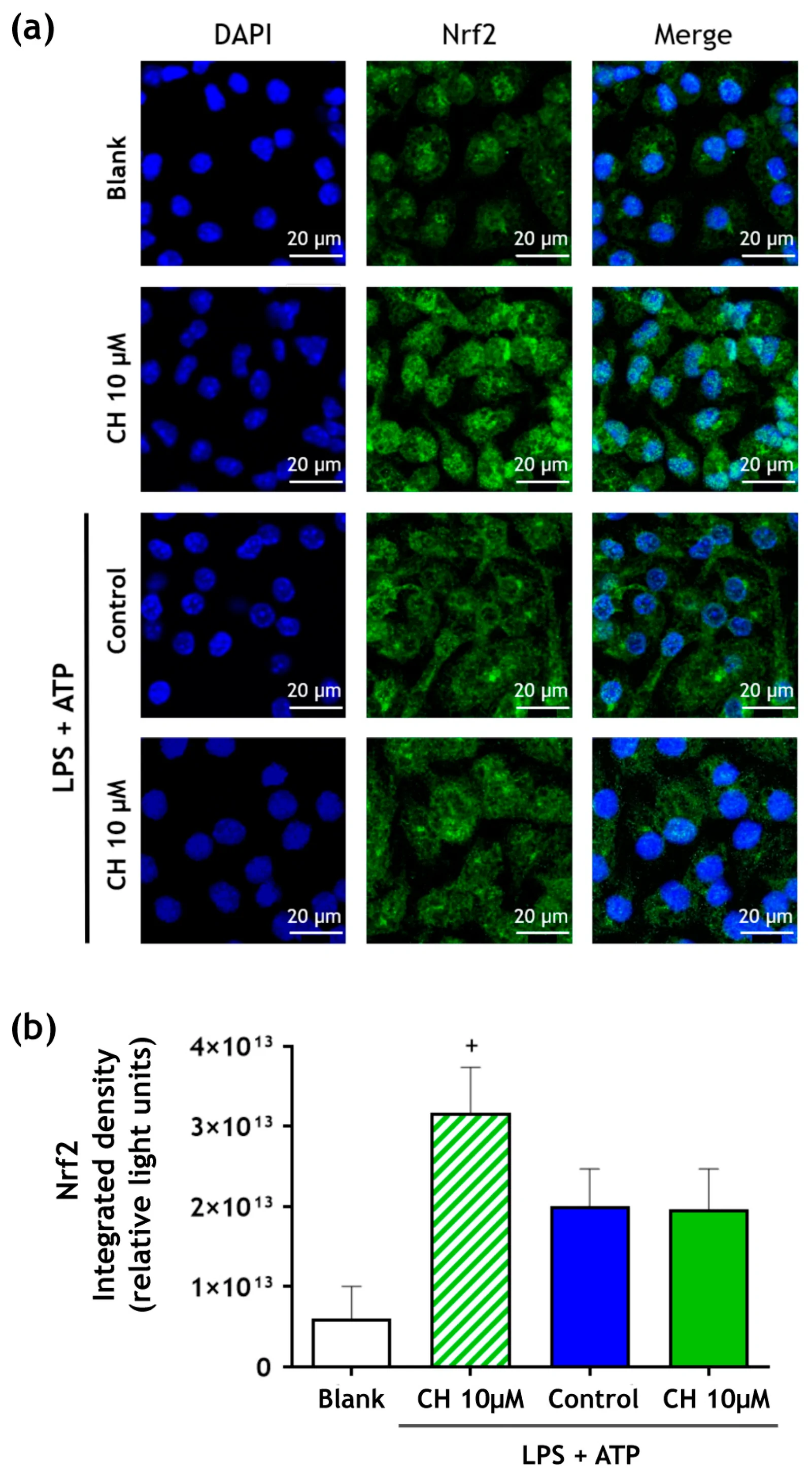
Effect of CH on Nrf2 nuclear translocation in peritoneal macrophages stimulated with LPS and ATP determined by immunofluorescence. (**a**) Representative image of Nrf2 nuclear translocation acquired using an Olympus FV1000 confocal microscope. Green fluorescence corresponds to Nrf2 staining and blue fluorescence corresponds to DAPI-stained nuclei. (**b**) Quantification of Nrf2 nuclear fluorescence intensity. Results are expressed as mean ± SD (*n* = 4). + *p* < 0.05 versus the blank group (one-way ANOVA followed by Tukey’s test). CH: 2,4,5-trimethoxy-2′-trifluoromethylchalcone; LPS: lipopolysaccharide; ATP: adenosine triphosphate.

To further explore the antioxidant effects of CH, nuclear translocation of Nrf2 was analyzed in peritoneal macrophages by immunofluorescence. Nrf2 is a transcription factor involved in the regulation of antioxidant and cytoprotective responses and has also been associated with modulation of inflammatory signaling pathways, including NLRP3 inflammasome activation [13]. Under basal conditions, Nrf2 staining was mainly distributed in the cytoplasm. Following CH treatment, Nrf2 immunostaining showed increased nuclear localization, particularly at the highest concentration tested (Figure 8).

### 3.2. Effect of CH on Calcium Pyrophosphate Dihydrate (CPPD) Crystal-Induced Mouse Air Pouch Model

#### 3.2.1. Effect of CH on Cell Infiltration and Myeloperoxidase (MPO) Activity on Air Pouch Exudates

**Figure 9:**
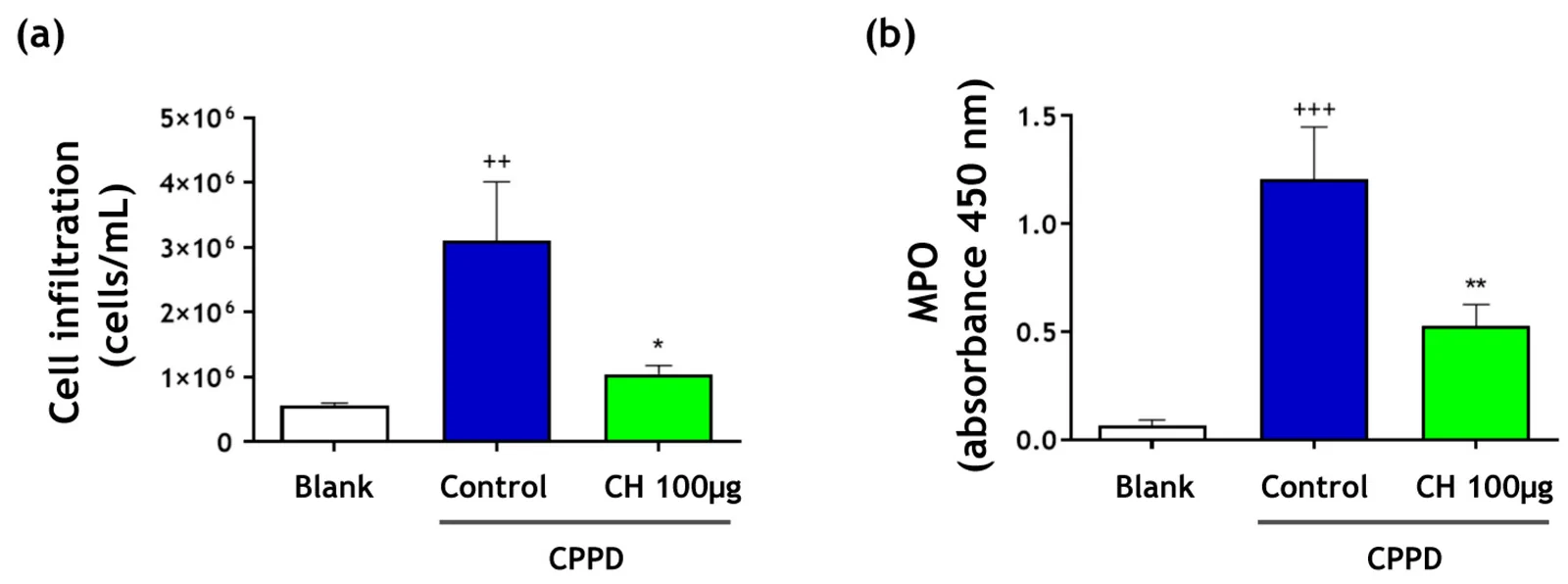
Effect of CH on leukocyte infiltration and myeloperoxidase (MPO) activity in the CPPD crystal-induced air-pouch model. (**a**) Leukocyte infiltration was assessed by determining total cell counts in air-pouch exudates using a Coulter counter. Results are expressed as cells/mL. (**b**) MPO activity was determined spectrophotometrically by measuring absorbance at 450 nm. Results are presented as mean ± SD (*n* = 6). ++ *p* < 0.01 and +++ *p* < 0.001 versus the blank group; * *p* < 0.05 and ** *p* < 0.01 versus the control group (one-way ANOVA followed by Tukey’s post hoc test). CH: 2,4,5-trimethoxy-2′-trifluoromethylchalcone; CPPD: calcium pyrophosphate dihydrate.

The anti-inflammatory effects of CH were subsequently evaluated in vivo using the CPPD crystal-induced air pouch model. Injection of CPPD crystals triggered a marked inflammatory response characterized by increased leukocyte infiltration and higher MPO activity in exudates compared with the basal group. As shown in Figure 9, CH significantly reduced CPPD-induced leukocyte infiltration and MPO activity in air-pouch exudates.

#### 3.2.2. Effect of CH on Pro-Inflammatory Cytokines on Air Pouch Exudates

**Figure 10:**
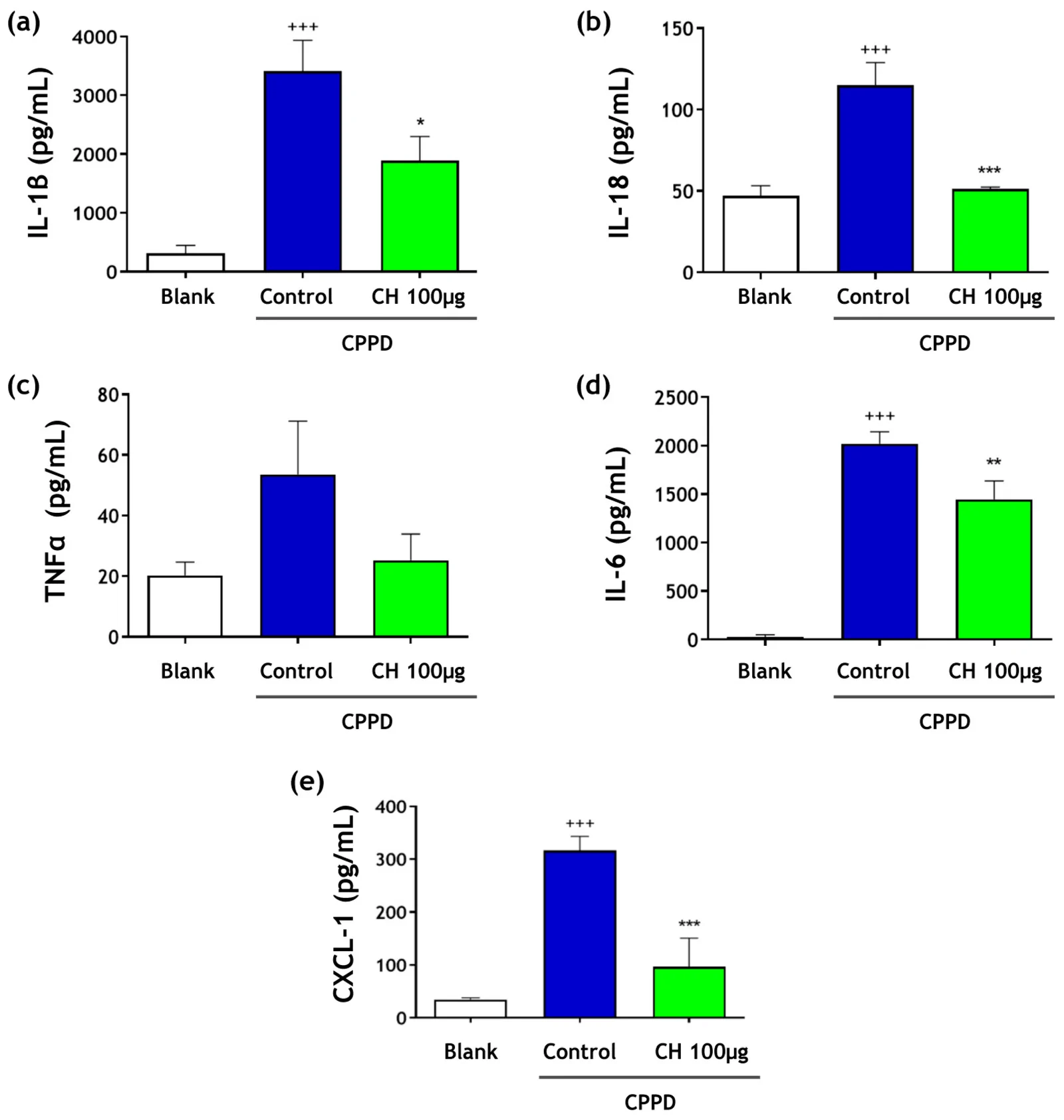
Effect of CH on the release of pro-inflammatory cytokines in the air-pouch model stimulated with CPPD crystals. Cytokines levels were determined by ELISA. (**a**) IL-1β; (**b**) IL-18; (**c**) TNF-α; (**d**) IL-6; (**e**) CXCL-1. Results are expressed as mean ± SD (*n* = 6). +++ *p* < 0.001 versus the blank group; * *p* < 0.05, ** *p* < 0.01, *** *p* < 0.001 versus the control group (one-way ANOVA followed by Tukey’s test). CH: 2,4,5-trimethoxy-2′-trifluoromethylchalcone; CPPD: calcium pyrophosphate dihydrate.

Inflammatory mediator production in air pouch exudates was evaluated by ELISA after CPPD crystal injection. Elevated levels of IL-1β, IL-18, TNF-α, IL-6 and CXCL-1 were detected in control animals stimulated with CPPD crystals. CH treatment reduced the levels of IL-1β, IL-18, IL-6 and CXCL-1, whereas TNF-α showed a non-significant downward trend (Figure 10a).

#### 3.2.3. Effect of CH on Caspase-1 and NF-κB Activation on Air Pouch Exudates

**Figure 11:**
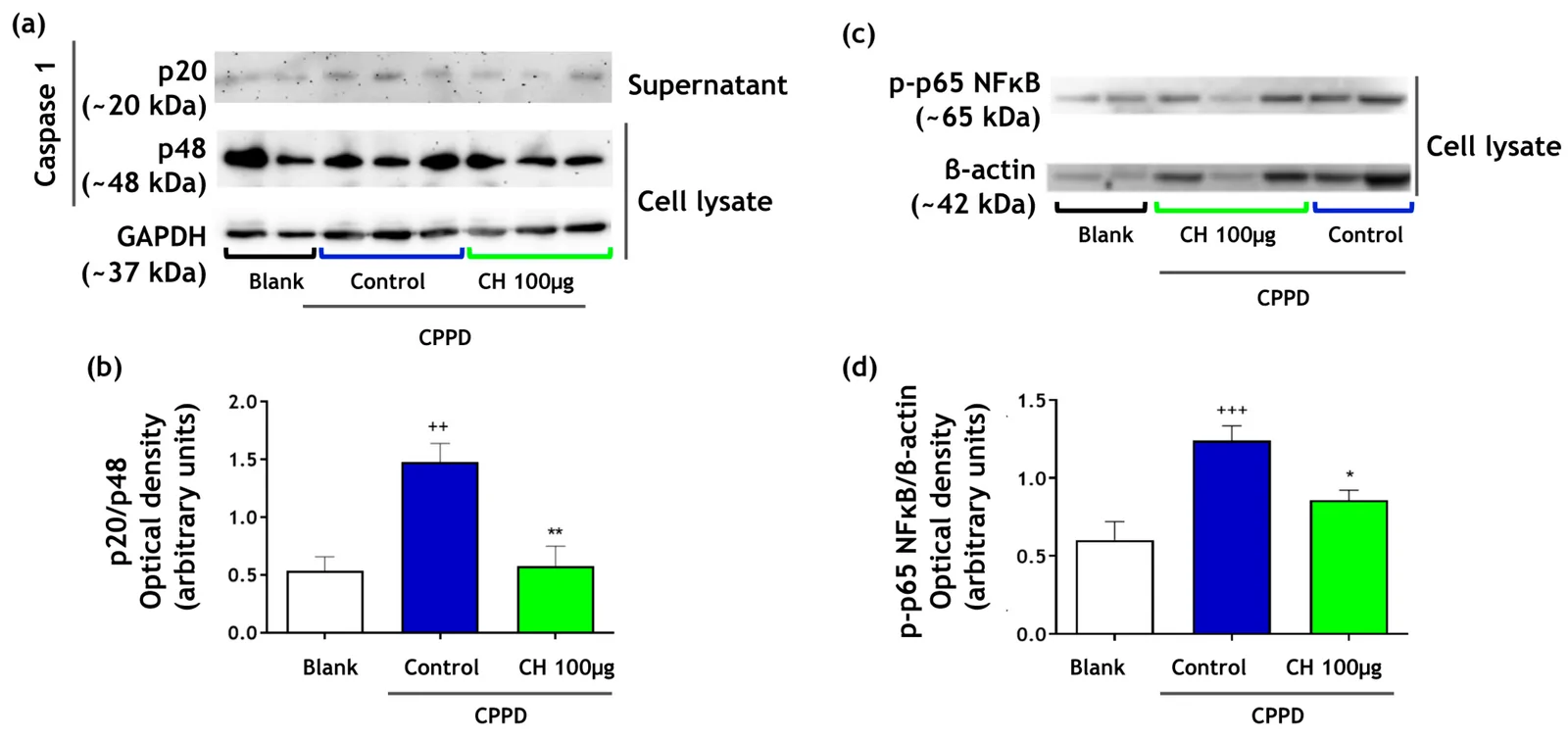
Effect of CH on caspase-1 activation (**a**,**b**) and p65 NF-κB subunit phosphorylation (**c**,**d**) in the air-pouch model stimulated with CPPD crystals. (**a**) Representative Western blot images showing p20 and p48 caspase subunits on protein extracts from supernatants and cell lysates from air pouch exudates. GAPDH was used as the loading control. (**b**) Densitometric analysis expressed as the p20/p48 ratio as arbitrary units of integrated optical density. (**c**) Representative Western blot images of p65- NF-κB expression in cell lysates of air pouch exudate. β-actin was used as the loading control. (**d**) p-p65- NF-κB/β-actin is expressed as arbitrary units of integrated optical density. Results are expressed as mean ± SD (*n* = 4–5). ++ *p* < 0.01, +++ *p* < 0.001 versus the blank group; * *p* < 0.05, ** *p* < 0.01 versus the control group (one-way ANOVA followed by Tukey’s test). CH: 2,4,5-trimethoxy-2′-trifluoromethylchalcone; CPPD: calcium pyrophosphate dihydrate.

To elucidate the mechanisms involved in the anti-inflammatory effects of CH in vivo, caspase-1 activation and NF-κB signaling were analyzed in air pouch exudates. CPPD crystals induced a marked increase in the active p20 caspase-1 subunit and in the p20/p48 ratio compared with basal conditions. In mice treated with CH, both parameters were reduced, indicating lower caspase-1 activation (Figure 11a,b).

NF-κB activation was evaluated by determining phosphorylation of the p65 subunit in cell lysates obtained from exudates. Compared with unstimulated animals, CPPD crystals produced a clear increase in p-p65 levels. This signal was less intense in samples from CH-treated mice, as confirmed by densitometric analysis (Figure 11c,d).

### 3.3. Effect of CH on Monosodium Urate (MSU) Crystal-Induced Gouty Arthritis Model

#### 3.3.1. Effect of CH on Plantar Edema, Myeloperoxidase (MPO) Activity and Cytokine Levels

To further evaluate the anti-inflammatory effects of CH in vivo, its activity was analyzed in a murine model of MSU-induced gouty arthritis. This model is characterized by neutrophil and macrophage recruitment together with activation of NF-κB and NLRP3 inflammasome-caspase-1 signaling, leading to the release of pro-inflammatory cytokines [7].

**Figure 12:**
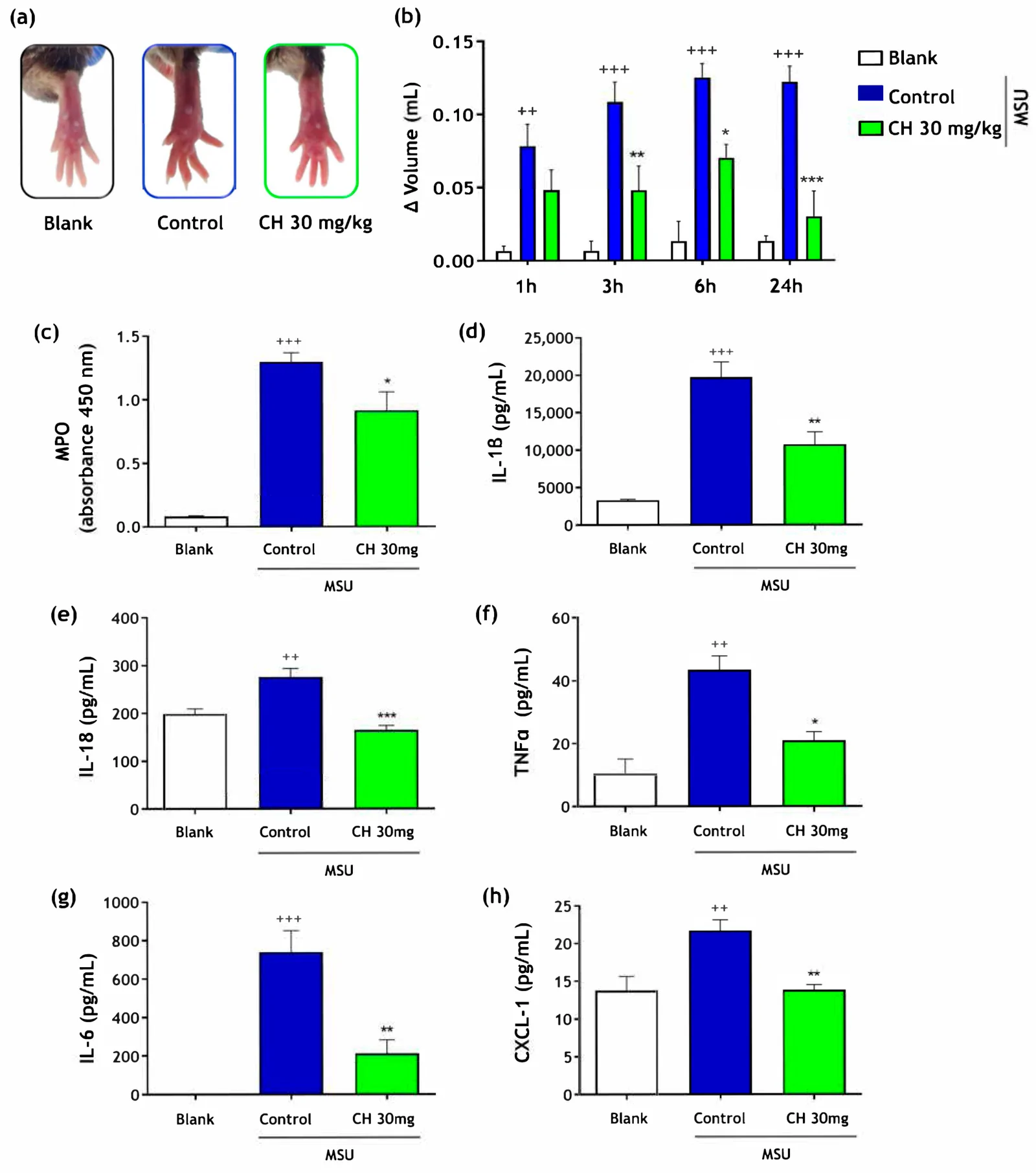
Effect of CH on paw edema (**a**,**b**), MPO activity (**c**) and IL-1β (**d**), IL-18 (**e**), TNF (**f**), IL-6 (**g**) and CXCL-1 (**h**) in the MSU-induced gout arthritis model. Paw edema was measured with a digital plethysmometer, MPO activity by spectrophotometry (optical density, 450 nm) in paw homogenates and cytokine levels by ELISA in supernatants. (**b**) Results are expressed as changes in paw volume (mL). Results are expressed as mean ± SD (*n* = 6). ++ *p* < 0.01 +++ *p* < 0.001 versus the blank group; * *p* < 0.05, ** *p* < 0.01, *** *p* < 0.001 versus the control group (two-way ANOVA followed by Bonferroni’s test). CH: 2,4,5-trimethoxy-2′-trifluoromethylchalcone; MSU: monosodium urate crystals.

Animals received CH (30 mg/kg) orally before MSU crystal injection into the paw. Paw edema was monitored, and paw homogenates were collected for the analysis of inflammatory parameters. The development of paw edema after MSU crystal injection was determined at different time points (1, 3, 6 and 24 h). Administration of MSU crystals induced a progressive increase in paw volume compared with the basal group. In animals treated orally with CH (30 mg/kg), edema formation was attenuated throughout the experimental period, with significant differences being observed at 3, 6 and 24 h (Figure 12a,b).

MPO activity was determined in paw homogenates as an indicator of neutrophil infiltration, since neutrophils recruited to the inflamed tissue actively participate in the response to MSU crystals. As shown in Figure 12c, paw homogenates from the control group displayed elevated MPO activity following MSU injection. Treatment with CH significantly reduced MPO activity.

Levels of IL-1β, IL-18, TNF-α, IL-6 and CXCL-1 were also determined in paw homogenates by ELISA. As shown in Figure 12d–h, MSU crystal injection markedly increased the levels of all inflammatory mediators analyzed compared with the basal group. Oral treatment with CH reduced cytokine production in inflamed paws, including IL-1β and IL-18, both associated with inflammasome activation. In addition, lower CXCL-1 levels were detected after CH treatment, in agreement with the reduction in MPO activity observed previously.

#### 3.3.2. Effect of CH on Caspase-1 Activation and NF-κB Signaling

Caspase-1 activation and NF-κB signaling were also evaluated in paw homogenates by Western blot. MSU crystals activate inflammatory pathways associated with the production of pro-inflammatory cytokines. For this purpose, the active caspase-1 p20 subunit and phosphorylation of the p65 NF-κB subunit were analyzed in inflamed paws.

**Figure 13:**
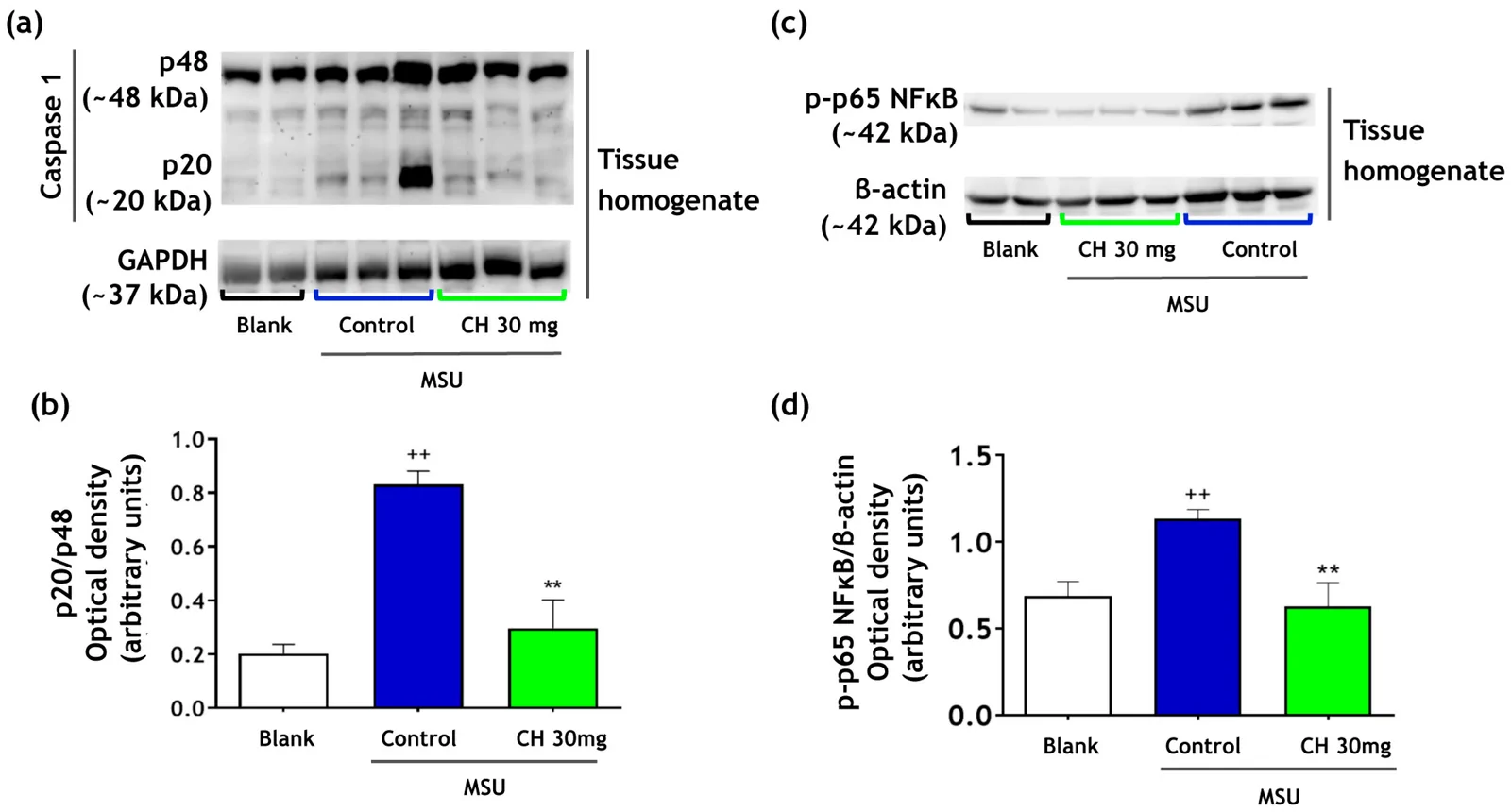
Effect of CH on caspase-1 activation (**a**,**b**) and p65 NF-κB subunit phosphorylation (**c**,**d**) in MSU gouty arthritis. (**a**) Representative Western blot images showing p20 and p48 caspase subunits on protein extracts from supernatants and cell lysates from paw homogenates. GAPDH was used as the loading control. (**b**) Densitometric analysis expressed as the p20/p48 ratio as arbitrary units of integrated optical density. (**c**) Representative Western blot images of p65- NF-κB expression in cell lysates of paw homogenate. β-actin was used as the loading control. (**d**) p-p65-NF-κB/β-actin is expressed as arbitrary units (a.u.) of integrated optical density (IOD). Results are expressed as mean ± SD (*n* = 4–5). ++ *p* < 0.01 versus the blank group; ** *p* < 0.01 versus the control group (one-way ANOVA followed by Tukey’s test).

Figure 12 shows that MSU crystal injection increased both p20 caspase-1 levels and p65 phosphorylation compared with the basal group. In contrast, lower p20 and p-p65 levels were detected in animals treated with CH (Figure 13). These findings were consistent with the reduction in IL-1β, IL-18, TNF-α and IL-6 levels previously observed in paw homogenates, indicating that CH attenuated both inflammasome activation and NF-κB signaling in this model.

## 4. Discussion

Crystal-induced arthropathies are characterized by activation of interconnected inflammatory and oxidative pathways involving innate immune responses, NLRP3 inflammasome activation, and neutrophil recruitment. Increasing evidence supports the contribution of oxidative stress and inflammasome-mediated signaling to the pathophysiology of gout and related crystal-induced inflammatory conditions [11]. In the present study, the chalcone derivative CH exerted anti-inflammatory and antioxidant effects in experimental models related to crystal-induced inflammation and gouty arthritis. CH reduced pro-inflammatory cytokine production, attenuated ROS generation, decreased NF-κB phosphorylation and caspase-1 activation, promoted Nrf2 nuclear translocation and reduced neutrophil infiltration in vivo. Importantly, these effects were consistently observed in LPS/ATP-stimulated macrophages as well as in CPPD crystal- and MSU-induced inflammatory models, suggesting coordinated modulation of interconnected redox-sensitive and inflammasome-related pathways.

Current evidence indicates that phagocytosis of MSU or CPPD crystals promotes activation of NF-κB signaling and the NLRP3 inflammasome, leading to the production of inflammatory mediators that amplify tissue inflammation [7,32]. NF-κB activation constitutes an important priming signal through induction of TNF-α, IL-6 and the inactive precursors of IL-1β and IL-18. In agreement with these mechanisms, CH reduced phosphorylation of the p65 NF-κB subunit together with decreased TNF-α and IL-6 production, suggesting attenuation of upstream inflammatory signaling events associated with innate immune activation.

Activation of the NLRP3 inflammasome plays a central role in crystal-induced inflammation. Caspase-1 activation promotes maturation and release of IL-1β and IL-18, cytokines involved in amplification of inflammatory responses and neutrophil recruitment during acute gout attacks [5,7]. Caspase-1 activation has additionally been associated with pyroptosis, an inflammatory form of programmed cell death characterized by membrane disruption and release of intracellular mediators [9]. In the present study, CH reduced IL-1β and IL-18 production together with lower caspase-1 activation in macrophages and in both in vivo models. Moreover, reduced LDH release in activated macrophages suggests attenuation of pyroptosis-associated membrane damage. Taken together, these findings support the contribution of inflammasome-mediated mechanisms in the anti-inflammatory effects of CH.

Extracellular ATP can activate NLRP3 inflammasome signaling through the purinergic P2X7 receptor, promoting caspase-1 activation and IL-1β release [33]. P2X7 signaling has also been implicated in ATP- and MSU-induced acute gouty inflammation [34]. Since ATP was used as the second stimulus for inflammasome activation in our macrophage model, a possible involvement of P2X7-dependent signaling in the anti-inflammatory effects of CH cannot be excluded and warrants further investigation.

Oxidative stress has also been closely associated with amplification of crystal-induced inflammatory responses. Crystal phagocytosis induces extracellular and mitochondrial ROS generation, which contributes to activation of redox-sensitive inflammatory pathways, including inflammasome-associated signaling [10,11]. In the present study, CH significantly reduced both extracellular and mitochondrial ROS production. Since ROS generation may act upstream of inflammatory signaling pathways, the antioxidant effects of CH could contribute, at least in part, to the reduction in NF-κB phosphorylation and caspase-1 activation observed in activated macrophages. These findings support a coordinated modulation of oxidative stress and inflammatory signaling pathways by CH.

Nrf2 is a key regulator of endogenous antioxidant responses and may negatively regulate inflammatory amplification associated with oxidative stress. Recent studies have suggested that activation of Nrf2 signaling may attenuate inflammatory responses in experimental gout [35]. Moreover, increasing evidence indicates the existence of functional crosstalk between Nrf2 and NF-κB signaling pathways, through which Nrf2 activation may limit oxidative stress-dependent inflammatory responses and inflammasome activation [36]. In agreement with this, CH increased Nrf2 nuclear translocation in activated macrophages. Although downstream antioxidant mediators were not evaluated, these findings are consistent with the antioxidant effects observed after CH treatment and may contribute, at least in part, to the reduction in ROS generation and inflammatory signaling detected in the present study.

Previous studies have demonstrated anti-inflammatory and antioxidant properties for several chalcone derivatives through modulation of inflammatory mediators, nitric oxide synthesis and oxidative stress-related pathways [17,18,22,23,24,25,26]. In particular, fluorinated chalcones have shown inhibitory effects on inducible nitric oxide synthase expression, nitric oxide production and NF-κB activation in activated macrophages [19,20]. The present findings extend these observations by showing that CH modulates pathways associated with crystal-induced inflammation, including ROS generation, inflammatory cytokine production and caspase-1-associated responses.

Neutrophils are major effector cells during acute crystal-induced inflammation and contribute to tissue injury through ROS production, protease release and amplification of cytokine responses [37]. Recruitment of neutrophils is strongly promoted by IL-1β and chemokines such as CXCL-1, contributing to amplification of inflammatory responses during crystal-induced arthritis [38]. In this context, CH reduced CXCL-1 production, leukocyte infiltration and MPO activity in both in vivo models, which was accompanied by attenuation of paw edema and inflammatory parameters in MSU-induced gouty arthritis.

An important aspect of the present study is the consistency of the pharmacological effects of CH across different experimental models. The compound was active not only in LPS/ATP-stimulated macrophages but also in CPPD-induced air pouch inflammation and MSU-induced gouty arthritis. Since CPPD and MSU crystals share partially overlapping inflammatory pathways involving oxidative stress, inflammasome-associated signaling and neutrophil recruitment, these findings support the potential relevance of CH in crystal-associated inflammatory conditions beyond a single experimental setting.

Some limitations of the present study should be acknowledged. Although the findings support modulation of inflammasome-associated pathways, direct assessment of NLRP3 inflammasome assembly or other inflammasome-related mediators was not performed. In addition, the in vitro experiments were conducted using LPS/ATP stimulation rather than crystal exposure, which may not fully reproduce the complexity of crystal-induced inflammatory responses. Likewise, downstream antioxidant mediators regulated by Nrf2 were not evaluated. Future studies should further clarify the molecular mechanisms involved in the effects of CH, including direct evaluation of NLRP3 inflammasome activation, the potential involvement of purinergic signaling pathways such as P2X7, and downstream antioxidant pathways regulated by Nrf2. In addition, chronic models of crystal-induced inflammation would help to better define the therapeutic relevance of this chalcone derivative.

## 5. Conclusions

In conclusion, the present study demonstrates that CH exerts anti-inflammatory and antioxidant effects in experimental models of crystal-induced inflammation and gouty arthritis. These effects were associated with reduced ROS generation, attenuation of NF-κB and caspase-1 activation, increased Nrf2 nuclear translocation and decreased neutrophil-driven inflammatory responses. Overall, the findings suggest that CH may represent a promising multitarget compound for the modulation of interconnected inflammatory and oxidative pathways involved in crystal-associated inflammatory diseases.

## Data Availability

All relevant data are presented in the manuscript. Raw data are available upon request from the corresponding authors.

## Abbreviations

The following abbreviations are used in this manuscript:

ATP	Adenosine 5′-triphosphate
a.u	Arbitrary units
BSA	Bovine serum albumin
CH	2,4,5-trimethoxy-2′-trifluoromethylchalcone
CPPD	Calcium pyrophosphate dihydrate
ECL	Enhanced chemiluminescence
ELISA	Enzyme-linked immunosorbent assay
FBS	Fetal bovine serum
HBSS	Hank’s balanced salt solution
IL	Interleukin
LDH	Lactate dehydrogenase
LPS	Lipopolysaccharide
MPO	Myeloperoxidase
MSU	Monosodium urate
MTT	3-(4,5-dimethylthiazol-2-yl)-2,5-diphenyltetrazolium bromide
NF-κB	Nuclear factor-kappa B
NLRP3	NOD-like receptor family pyrin domain containing 3
Nrf2	Nuclear factor erythroid 2-related factor 2
PBS	Phosphate-buffered saline
RLU	Relative light units
RPMI	Roswell Park Memorial Institute medium
ROS	Reactive oxygen species
SD	Standard deviation
TMB	3,3’,5,5’-tetramethylbenzidine
TNF	Tumor necrosis factor
TPA	12-O-tetradecanoylphorbol-13-acetate
